# Effect of the Simulated Disinfection by Microwave Energy on the Impact Strength of the Tooth/Acrylic Resin Adhesion

**DOI:** 10.2174/1874210600802010013

**Published:** 2008-01-22

**Authors:** Rafael L.X. Consani, Marcelo F. Mesquita, Marinaldo H. Zampieri, Wilson B. Mendes, Simonides Consani

**Affiliations:** 1 Department of Prosthodontics and Periodontics, Piracicaba Dentistry School, State University of Campinas, Brazil;; 2 Department of Prosthodontics and Periodontics, Piracicaba Dentistry School, State University of Campinas, Brazil;; 3 Piracicaba Dentistry School, State University of Campinas, Brazil; 4 Department of Clinics, Itauna University Dentistry School, MG, Brazil; 5 Department of Restorative Dentistry, Piracicaba Dentistry School, State University of Campinas, Brazil.

**Keywords:** Microwave disinfection, impact strength, tooth/acrylic resin adhesion, glossy ridge lap retention

## Abstract

The objective of this study was to determine the effect of simulated microwave disinfection on the tooth/acrylic resin impact strength. Acrylic molar teeth with a wax stick attached to the ridge lap were included in brass flasks. Specimens were made with Classico thermopolymerized acrylic resin, according to the groups: 1 and 5 - tooth with no treatment (control); 2 and 6 – tooth bur abrasion; 3 and 7 – tooth bur retention; and 4 and 8 – tooth monomer etch. Eighty specimens (n=10) were polymerized in bath cycle at 74^º^C for 9 hours and deflasked after flask cooling. Specimen from groups 2, 4, 6 and 8 was submitted to simulated microwave disinfection in a microwave oven at 650W for 3 minutes. Impact strength test was performed with an Otto Wolpert-Werke machine (Charpy system) with an impact load of 40 kpcm. Fracture load value was transformed into impact strength as a function of the bond area (kfg/cm^2^). Collected data were submitted to ANOVA and Tukey’s test (α=.05) and results indicate that the simulated microwave disinfection decreased the impact strength in all treatments.

## INTRODUCTION

In addition to the contamination caused during manufacture or manipulation, or by patients, prostheses can be contaminated by microorganisms during clinical use. In an effort to eliminate or decrease cross-contamination, prostheses should be disinfected with suitable chemical solutions [1,2].

Studies have shown that sterile prostheses are contaminated during polishing with pumice slurry or by microorganisms transferred from other prostheses during cloth wheel polishing procedures used in common laboratory practice [3,4]. 

Methods for prosthesis chemical disinfection have been suggested by many authors to avoid the cross-contamination promoted by pathogenic agent dissemination, including the use of glutaraldehyde, sodium hypochlorite, iodoform, chlorine dioxide or alcohol solutions [5-7].Chemical disinfection, however, does have some disadvantages, such as prosthesis staining and oral tissue reactions in the patient [8-9]. 

To minimize the disadvantages of chemical disinfection, the use of microwave energy has been suggested as a simple alternative to prosthesis disinfection, with lower operational costs and ease of use [10]. Microwave energy was originally used for the thermally-activated acrylic resin polymerization [11,12];however, the irradiation of resilient linings and acrylic resins immersed in water in a domestic microwave oven effectively sterilizes specimens contaminated by the fungi [8], *Candida albicans* or *Staphylococcus aureus* [9].

Due to the probability that the acrylic resin denture base is contaminated both internally and externally [7], the use of microwave energy has been recommended as an ideal method for disinfection [8,9]. 

The effects of microwave disinfection on hardness or flexural strength of the acrylic resin [10],and on the dimensional accuracy of the denture base [13] have shown that the results were not significantly altered by the disinfection procedure. Microwave post-polymerization irradiation can also be an effective method for increasing the flexural strength of denture relining resins [14].A recent study demonstrated that simulated disinfection by microwave energy improved denture base adaptation when the traditional clamp flask closure method was used [15].

Fracture of the tooth/denture base bond may be caused by excessive stress or by fatigue. A poor laboratory technique that impedes a satisfactory bond between tooth and base resin can also be responsible for many failures [16].Imperceptible traces of wax, not removed by methods of wax elimination, seem to be the principal contaminant and have a highly significant detrimental effect on the bond, causing consequent adhesive failure [17,18]. Changes in the surface of the glossy ridge-lap surface by grooving or retention do not make a significant difference when compared with unmodified surfaces [19,20], while a significant increase in bond strength is obtained when suitable bonding agents are applied [21].

Studies have been developed with the purpose of demonstrating the influence of mechanical retentions on glossy ridge laps [22,23], and the monomer etching effect on the unmodified ridge laps [24-25] in the adhesive strength between tooth and denture base. In addition, few studies had been developed with the aim of characterizing the effect of microwave disinfection on the impact strength of the tooth/base resin adhesion, a condition that may modify the denture’s durability during oral use. 

The purpose of the present study was to verify the effect of simulated microwave disinfection on the impact strength of the tooth/acrylic resin adhesion, when the glossy ridge laps were unmodified, abraded, grooved or etched by monomer. The research hypothesis tested was that the tooth/resin bond would be adversely affected by simulated microwave disinfection, independently of the different treatments to the glossy ridge laps. It may be speculated that the stiffness of the acrylic resin irradiated by microwave energy could be increased, resulting in decreased bond strength in the tooth-resin adhesion. 

## MATERIALS AND METHODS

### Specimens

Wax rectangular mold patterns were poured into each traditional brass flask (Safrany; Safrany Metallurgy, Sao Paulo, SP, Brazil) with type III dental stone (Herodent; Vigodent, Rio de Janeiro, RJ, Brazil), proportioned and manipulated according to the manufacturer’s instructions. After wax pattern removal, the rectangular stone mold was filled with a layer of laboratory silicone putty (Zetalabor; Zhermack, Rovigo, Italy) hard type (85 shore A). Identical shape and size model 34L acrylic molar teeth (Biotone; Dentsplay, Petrópolis, RJ, Brazil), attached with a wax stick (6mm in diameter and 20mm in length) at the ridge lap surface, were partially placed into the silicone layer. The resultant wax stick and attached tooth set was covered with laboratory silicone putty (Zetalabor; Zhermack, Rovigo, Italy). After dental stone isolation with petroleum jelly, the flask was completely poured with type III dental stone (Herodent; Vigodent, Rio de Janeiro, RJ, Brazil) and pressed in a hydraulic press (Linea H; Linea, Sao Paulo, SP, Brazil)for 1 hour. 

**Fig. (1):**
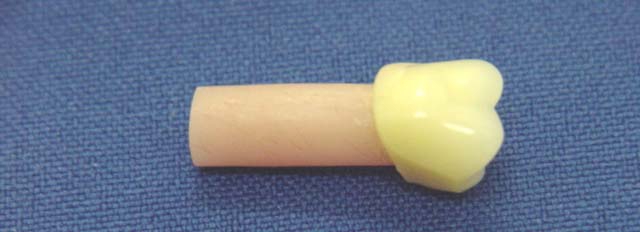
–Specimen for the impact strength test.

After pressing, the tooth/wax stick set was deflasked and the wax stick removed from the tooth. The tooth was brushed with hot water and liquid household detergent (Bombril; Bombril-Cirio, São Paulo, SP, Brazil) solution to eliminate the wax sticks residues and rinsed with running water. Five specimens (Fig. **1**) were made in each flask with the tooth attached to the denture base acrylic resin, proportioned and manipulated according to the manufacturer’ instructions, using one of the following protocols:

1– tooth without treatment (control); 2- glossy ridge lap grinding with bur; 3- glossy ridge lap grooving with bur; 4- glossy ridge lap etched by monomer applied for 30 seconds with a small brush before packing [26]. 

The specimens of the protocols 5, 6, 7 and 8 were made similarly to protocols 1, 2, 3 and 4 with exception they were submitted afterwards to simulated microwave disinfection in a domestic microwave oven (Continental; Continental Domestic Lines, Manaus, AM, Brazil) for 3 minutes at 650W [9]. For this procedure the specimens were immersed individually in 150mL of distilled water in a glass container. The specimen was removed from the glass container with a tweezers after water cooling at room temperature, and dried with air before impact strength test. 

The acrylic resin (Classico; Classico Dental Products, Sao Paulo, SP, Brazil) was prepared using a solution with a ratio of 35.5g polymer to 15mL monomer, according to manufacturer’s instructions. The flasks were placed in traditional clamps after final pressing in a hydraulic press(Linea H; Linea, Sao Paulo, SP, Brazil) under a load of 1,250 kgf for 5 minutes. Eighty specimens (n=10) were conventionally packed, polymerized in a water bath at 74^0^C for 9 hours in a polymerizing unit (Termotron; Piracicaba, Sao Paulo, SP, Brazil)), deflasked after flask cooling at room temperature, and finished with abrasive stones. 

The bond impact strength test was measured in the non-disinfected (control) and submitted to simulated microwave disinfection specimens in an impact machine (Wolpert; Otto Wolpert-Werke, Ludwigshafen/Rhein, Germany), using the Charpy system with an impact load of 40 kpcm. The impact strength (kgf/cm^2^) was calculated as a function of the load applied at the moment of specimen failure (kpcm) and tooth/resin bonding area, using the equation:

IS = F / π.r^2 ^where:

IS = Impact strength (kgf/cm^2^).

F = Failure load (kpcm).

π.r^2 ^= tooth/resin bonding area; where: π = 3.1416 and r^2 ^= 0.09cm^2^; thus, 0.09x3.1416=0.28cm^2^. 

Observation of the failure mode after impact strength test was under an optical microscope (EMZ-TR; Meiji Thecno Co., Tokyo, Japan), with 1.5x magnification.

### Statistical Analysis

Data were submitted to 2-way analysis of variance (SANEST – Statistical Analysis System), considering 2 factors (ridge lap treatment and simulated microwave disinfection) and their interactions. Since same-factor interactions were significant, differences were submitted to multiple comparison testing (Tukey HSD test at α=.05).

## RESULTS

Two-way ANOVA (Table **1**) revealed significant differences in the tooth/resin bond impact strength for the different treatments (p<.00001), and simulated microwave disinfection (p<.00001), and their interactions (p<.00001).

Impact strength means following the use, or not, of microwave disinfection are shown in the Table **2**. In the non-disinfected specimens, control and monomer etching presented lower means, but were statistically different when compared to bur abrasion and bur grooving treatments (no statistically significant difference for these latter treatments). In the disinfected specimens, control and monomer etching presented the lowest means and were statistically significantly different when compared to bur abrasion and bur grooving treatments, both with statistically significant difference. When the non-disinfected and disinfected specimens were compared, all treatments demonstrated means with statistically significant difference and were lower in the specimens submitted to disinfection.

In all groups, the predominant failure was mixed (adhesive, and cohesive in the acrylic resin). Mixed failure (adhesive, and cohesive in the tooth) was only showed in 3 specimens, and not any adhesive failure was observed.

## DISCUSSION

In the present *in vitro* study, the research hypothesis that the tooth/resin bond would be adversely affected by simulated microwave disinfection, independently of the different glossy ridge lap treatments was accepted. The 2-way ANOVA revealed significant difference in the impact strength for the different ridge lap treatments and microwave disinfection. The interactions between treatment and disinfection were also significant (Table **1**). Independently of the microwave disinfection, control and monomer etched specimens demonstrated lower means that were statistically different when compared to bur abrasion and bur grooving treatments, both showing no statistically significant difference (Table **2**). 

Although wax appears to be the principal contaminant and cause of adhesive failure of the tooth-base resin bond [17,18], the denture base material and denture tooth selected may influence the tensile bond strength of the tooth to the base [27]. Physical modification by retention grooves of different shapes on the tooth surface had no significant effect on the bond strength [22, 28], and grinding of the tooth may only be beneficial to bonding in the absence of wax traces [19]. For preventing such failures, the use of modern synthetic detergents that effectively remove all traces of wax is necessary [17]. Detergent solution was used in this study to remove the traces of wax from the teeth. Thus, it may be speculated that the statistical significance showed may be due to the different treatments performed on the tooth ridge laps. 

Previous studies have shown that painting with monomer or grinding the ridge lap of the tooth before packing did not seem to improve the adhesion to the resin base [18,25]. Conversely, the present investigation showed that findings from these treatments were not similar. So, results from control and monomer etched specimens were statistically similar, and bur abrasion as well as bur grooving specimens significantly improved the tooth/resin bond (Table **2**). 

In the present study, the similarity of the impact strength values for control and monomer etched specimens was probably due to the cross-linking added to the methylmetacrylate denture teeth for improving surface hardness and abrasion resistance [26]. This procedure, however, results in decreased bond strength as compared to acrylic resin teeth with no cross-linking [29]. It was showed that when the hardness of the tooth is increased, the bonding strength between the tooth and the denture base decreases [30]. 

Chemical interaction of the acrylic resin and ridge lap portion of the tooth influences the bond strength; however, monomer etch had a dramatic effect, decreasing the failure load when the tooth was painted with monomer alone [24] or when a highly cross-linked denture tooth was used [26]. Different tooth surface modifications resulted in significantly different bond strengths [26]. The mechanical retention, in the form of a grind or groove placed in the ridge lap of the tooth, increased the impact strength values (Table **2**). The finding of this investigation is in agreement with classic studies evaluating the bonding of the denture teeth to the acrylic resin base, where mechanical retention was used on the ridge lap [20,22,24,31]. Thus, bur abrasion and bur grooving presented impact strength values that were not statistically significantly different.

It has been suggested that a tooth rough surface may trap wax residues, decreasing the bond strength [18], whereas an evaluation of the fractured sample showed that the resin mass does not penetrate into the groove made on the ridge lap of the tooth [31]. Within the limitations of this study, the results presented do not agree with the findings of these cited authors. 

Earlier study showed that a visual inspection of the fractured areas did not show the fracture mode, suggesting debonding due to deficient resin mass penetration into the irregularities of the ridge lap. In these treatments, better bond strength was attributed to greater surface area and better penetration of the resin mass to the tooth irregularities [31]. Bur grooving may decrease or increase the bonding strength in similar chemically acrylic resins, and the reason for this apparent controversy is difficult to determine [26]. 

Table **2** also shows the influence of microwave disinfection on the tooth/acrylic resin impact strength. Control and monomer etched specimens presented lower means that were statistically different when compared to bur abrasion and bur grooving treatments, both with statistically significant difference. Simulated microwave disinfection led to statistically different values of impact strength for the different mechanical treatments. The highest result, for bur ground specimens, was probably due to the different level of irregularities caused by these procedures. 

Regions of the denture base with minimal restriction to additional polymerization shrinkage promoted by microwave energy showed better adaptation to the stone cast [15]. By analogy, it may be theorized that a similar phenomenon occurred on the flat surface of the ridge lap, improving the mechanical retention by resin shrinkage with minimal restriction in the adhesion area. Conversely, the additional contraction of the acrylic resin decreased the retention in the depth area of the groove. This decrease in retention is due to the stress induced into the groove by the additional microwave energy polymerization that is afterwards released. The internal stress released by the microwave disinfection may also cause distortion of the denture resin base [10,12]. 

When the non-disinfected and disinfected specimens were compared, all ridge lap treatments showed means with statistically significant differences, and all means were lower than those of specimens submitted to disinfection (Table **2**). Although flexural strength is not significantly altered by microwave energy, there was a small increase in the acrylic resin hardness. A possible explanation for the increased hardness resulting from microwave disinfection might be the lack of water plasticizing effect occurring in the microwave radiated specimens [10]. Conversely, in this study, the specimens were adversely affected by simulated microwave disinfection, decreasing the bond strength values between tooth and acrylic resin.

Irradiation by microwave energy generates heat inside the acrylic resin [11], increasing the degree of conversion of autopolymerizing acrylic resins [32] due to a decreased level of residual monomer [14]. Thus, it may be speculated that the stiffness of the acrylic resin irradiated by microwave energy was increased, resulting in a decreased cohesive strength in the irradiated specimens. This decreased strength was evident in all specimens irradiated by microwave energy. 

As specified above for the non-disinfected specimens, greater impact strength was shown in the mechanical retention of the irradiated specimens. Due to the material volume trapped inside the ridge lap groove, the deleterious effect of the decreased cohesive strength on the bonding was more evident than the surface roughness of the ground specimens. 

The results of this study show that simulated microwave disinfection may be deleterious to tooth/base resin adhesion. The decrease in bond strength may displace the tooth from the base, either by masticatory load or due to accidental dropping during denture cleaning. Although attempts were made to characterize the effect of microwave irradiation on the tooth/resin bond, this study is limited in predicting the effect of other variables involved in the investigation. Further studies are necessary to evaluate whether the effect of microwave energy on tooth-resin impact strength may be deleterious to the denture base adaptation and stability in oral use. 

The failure resulting from the impact strength test was predominantly mixed (adhesive, and cohesive in the acrylic resin). This fact signifies that the cohesive strength of the tooth is greater when compared to the acrylic resin cohesive strength, and the failure of the bond will be probably in the acrylic resin base when the denture is in use. 

## CONCLUSION

Within the limitations of this *in vitro *study, the results suggest that the simulated microwave disinfection significantly decreased the tooth/resin impact strength. Additionally, mechanical retention improved the impact strength when compared to the control and monomer etched treatments; however, the impact load was lower in the disinfected specimens.

## Figures and Tables

**Table 1.** Results of Two-Way ANOVA Statistical Analysis

Variation Cause	*df*	Sum of Squares	Mean Square	F	P
Treatment (T)	3	7115.638	2371.879	215.062	.00001
Disinfection (D)	1	2699.327	2699.327	244.752	.00001
T x D	3	725.346	241.782	21.922	.00001
Error	72	794.072	11.028		
Total	79	11334.384			

General mean = 19.578; variation coefficient = 16.963%

**Table 2.** Impact Strength Means (kgf/cm^2^) and SD in Rela-tion to Microwave Disinfection Treatment

Treatment	Microwave Disinfection	
	Non-Disinfected	Simulated Disinfection
Control	12.31 ± 0.69 b A	7.73 ± 1.50 c B
Bur abrasion	39.45 ± 3.34 a A	23.06 ± 4.37 a B
Bur grooving	35.77 ± 1.89 a A	17.10 ± 4.99 b B
Monomer etch	14.00 ± 2.70 b A	7.17 ± 4.39 c B

Means followed by different lowercase letters in each column and capital letter in each row differ significantly by Tukey’s test (p<.05).
